# Identification of a novel *CLCN2* homozygous variant in a man with leukoencephalopathy and infertility: a case report and literature review

**DOI:** 10.3389/fgene.2026.1761205

**Published:** 2026-02-25

**Authors:** Lijia Yu, Weiqing Jiang, Li Cao, Zhi Geng, Jingjiong Chen

**Affiliations:** 1 Department of Neurology, Shanghai Sixth People’s Hospital Affiliated to Shanghai Jiao Tong University School of Medicine, Shanghai, China; 2 Department of Neurology, Shanghai Tongji Hospital, School of Medicine, Tongji University, Shanghai, China

**Keywords:** chloride ion channel, ClC-2, *CLCN2*, leukoencephalopathy with ataxia, loss-of-function variant

## Abstract

Leukoencephalopathy with ataxia (LKPAT), also known as *CLCN2*-related leukoencephalopathy, is a rare autosomal recessive disorder caused by pathogenic variants in *CLCN2,* which encodes ClC-2, a ubiquitously expressed chloride channel protein. However, due to high variability in clinical presentation leading to underdiagnosis, very few cases have been reported since its first description in 2013. The prevalence and genotype–phenotype correlations of LKPAT remain unclear, as do the pathogenic mechanisms of *CLCN2* variants. In this study, we reported a Chinese man who presented with dizziness, weakness of the left lower limb, and mild cerebellar ataxia. Notably, the patient had a history of azoospermia. Brain MRI showed symmetrical and confluent white matter abnormalities with hypointense signals on T1-weighted images and hyperintense signals on T2-weighted images. In this patient, a novel biallelic missense variant p.A506V was identified in *CLCN2*. Through *in silico* analysis, we observed that substitution of A506 with V506 altered hydrogen bond formation at chloride-binding sites. In addition, the A506V variant impacted the interaction of ClC-2 with GlialCAM, a ClC-2 auxiliary subunit that can physically bind ClC-2 and regulate its biophysical properties and subcellular localization in glial cells. Furthermore, we reviewed the literature and identified potential genotype–phenotype correlations in *CLCN2*-related diseases. Our results highlight the need for *CLCN2* genetic analysis to establish a definitive diagnosis when strong diagnostic clues are present. This study expands the genotypic spectrum of LKPAT, indicates the potential pathogenesis of the *CLCN2* A506V variant, and provides valuable insights into further investigation into therapeutics of *CLCN2*-related leukoencephalopathy.

## Introduction

Leukoencephalopathy with ataxia (LKPAT) is a rare autosomal recessive disorder clinically characterized by atypical neurological features including mild ataxia, cognitive impairment, psychiatric symptoms, headaches, and spasticity, along with a variable clinical presentation, which may include mild visual impairment, auditory abnormalities, and male infertility. However, specific findings on brain MRI have been recognized as symmetrical white matter signal abnormalities in the posterior limbs of the internal capsules, midbrain cerebral peduncles, and middle cerebellar peduncles. LKPAT, also known as *CLCN2*-related leukoencephalopathy, is associated with pathogenic variants in the *CLCN2* gene encoding ClC-2, a ubiquitously expressed chloride channel protein involved in the regulation of brain ion and water homeostasis (Jentsch and Pusch, 2018). To date, only 31 pathogenic variants of *CLCN2* have been identified in 48 affected individuals from 45 unrelated families worldwide (Depienne et al., 2013; Di Bella et al., 2014; Hanagasi et al., 2015; Giorgio et al., 2017; Zeydan et al., 2017; Hoshi et al., 2019; Guo et al., 2019; Ozaki et al., 2020; Parayil Sankaran et al., 2020; Ngo et al., 2020; Shi et al., 2021; Ben Mohamed et al., 2022; Abreu et al., 2023; Almasoudi et al., 2023; Holla et al., 2023; Wu et al., 2023; Cheng et al., 2023; Nóbrega et al., 2024; Garg et al., 2024; Zhou et al., 2025; Ohira et al., 2024; Orimo et al., 2025; Bavdhankar et al., 2025). In this study, we report a novel biallelic missense variant in *CLCN2* in an individual with leukoencephalopathy and infertility and conduct *in silico* analysis to identify the effect of this variant on the ClC-2 protein structure. Furthermore, by including data from previously published *CLCN2*-related cases, we present a cumulative and comprehensive clinical characterization and describe the genotype–phenotype features of *CLCN2*-related diseases.

## Materials and methods

### Subjects

A Chinese family was recruited through the Department of Neurology, Shanghai Sixth People’s Hospital, affiliated with Shanghai Jiao Tong University School of Medicine. Detailed clinical information, family history, and clinical characteristics were collected through interviews by two experienced neurologists. Informed consent was obtained from the patient, and this study was approved by the Ethics Committee of Shanghai Sixth People’s Hospital.

### Targeted next-generation sequencing

Variant screening of 320 candidate genes associated with leukodystrophies and genetic leukoencephalopathies was performed in the patient by targeted next-generation sequencing (NGS), as previously described (Zhou et al., 2017). In brief, all exons and flanking intronic sequences of the candidate pathogenic genes were captured using customized capture arrays (MyGenostics, Beijing, China) and sequenced by high-throughput sequencing on a Illumina HiSeq 2000 analyzer (Illumina, San Diego, CA, United States). Then, the reads were mapped by SOAPaligner (http://soap.genomics.org.cn/soapaligner.html) and Burrows–Wheeler Aligner (BWA) (http://bio-bwa.sourceforge.net/bwa.shtml). SNPs and variants were detected by GATK and SOAPsnp (http://soap.genomics.org.cn/soapsnp.html). Non-synonymous variants with minor allele frequencies (MAFs) of less than 0.05 were further evaluated for pathogenic variants. The MAFs of the candidate variants were obtained from the ExAC (https://exac.broadinstitute.org/) and gnomAD databases, which include data from over 60,000 individuals. Candidate pathogenic variants were verified by Sanger sequencing of both forward and reverse strands.

### Analysis of repeat expansions in *FMR1* and *NOTCH2NLC*

Genomic DNA was isolated from whole blood using standard methods. As previously reported (Chen et al., 2025), *FMR1* and *NOTCH2NLC* repeat expansions were measured by repeat‐primed PCR (RP‐PCR), and amplified products were detected by capillary electrophoresis (3730xl Genetic Analyzer, Thermo Fisher Scientific, Waltham, MA). Data were analyzed using GeneMarker. A saw‐tooth tail pattern in the electropherogram was considered to be the disease‐associated repeat expansion.

### Bioinformatics

We evaluated the possible pathogenic effect of the newly identified *CLCN2* missense variant using the prediction programs PolyPhen-2 (http://genetics.bwh.harvard.edu/pph2/), SIFT (Sorting Intolerant From Tolerant, http://sift.jcvi.org/), BayesDel_noAF, REVEL, and VEST4. Probabilistic scores of PolyPhen-2 were set at >0.85, 0.15–0.85, and <0.15 for probably damaging, possibly damaging, and benign, respectively. The SIFT score threshold for deleterious variants was set at ≤0.05.

### Molecular modeling and *in silico* analysis

The predicted protein model of mutant ClC-2 was built using the SWISS-MODEL server (http://swissmodel.expasy.org/) based on the crystal structure of the human ClC-2 (PDB code: 7XF5). We displayed the crystal structural model of human ClC-2 using PyMOL (https://pymol.org/2/). To analyze the interaction between mutant ClC-2 and GlialCAM, prediction of the protein docking model was performed using the ZDOCK online server (https://zdock.umassmed.edu) (Pierce et al., 2014). The ZDOCK program automatically generated the top 10 predictions. A higher score for the docking pose means higher binding ability (Wang et al., 2024). In this study, the pose with the highest ZDOCK score was visualized and analyzed using PyMOL.

## Results

### Clinical characteristics of the affected individual

The patient, a 48-year-old male, first presented with dizziness at the age of 45. He had no vertigo or tinnitus. In the following 3 years, repeated attacks of dizziness had been slowly progressive, and recently, he suffered from weakness in the left lower limb. He could walk, but he ran with fatigue in his left leg. Neurological examination revealed weakness and wasting in the left lower limb (Medical Research Council scale 4–5). Mild cerebellar ataxia, including bilateral dysmetria and intention tremor, was observed. There were no signs of gait ataxia, nystagmus, or dysarthria. Muscle tone and deep tendon reflexes of the extremities were normal. Additionally, bilateral positive Babinski signs were identified in the patient. The Mini-Mental State Examination (MMSE), one of the most widely used cognitive screening instruments, was administered for the initial cognitive evaluation of the patient, who had fewer than 6 years of formal education (Crum et al., 1993; Folstein et al., 1975). The MMSE score was 24. Notably, the patient had a history of azoospermia tested by semen analysis during his childbearing age. In addition, he was born to consanguineous parents.

**FIGURE 1:**
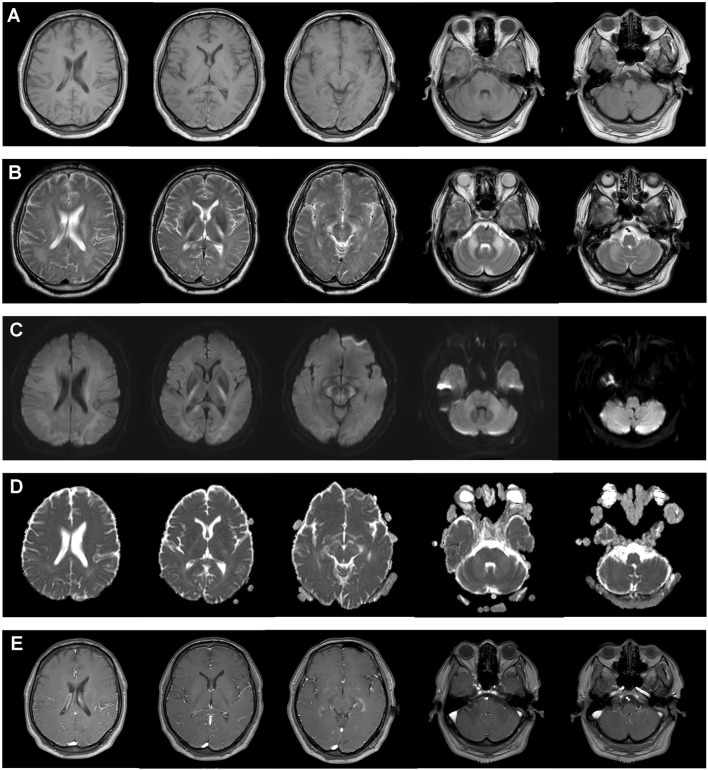
Brain magnetic resonance imaging of the patient. Symmetrical and confluent white matter abnormalities with hypointense signals on T1-weighted images **(A)** and hyperintense signals on T2-weighted images **(B)** were observed in the posterior limbs of the internal capsules; midbrain cerebral peduncles; middle cerebellar peduncles; bilateral frontal, parietal, and occipital white matter; the splenium of the corpus callosum; and cerebellar white matter. High signal intensity on diffusion-weighted imaging **(C)** without restriction on the apparent diffusion coefficient **(D)** was observed in the involved areas. **(E)** No contrast enhancement was observed in the post-gadolinium T1-WI sequence.

Brain magnetic resonance imaging (MRI) of the patient showed symmetrical and confluent white matter abnormalities with hypointense signals on T1-weighted images and hyperintense signals on T2-weighted images in the posterior limbs of the internal capsules, midbrain cerebral peduncles, and middle cerebellar peduncles (Figures 1A,B). Signal abnormalities were also present in the bilateral frontal, parietal, and occipital white matter; the splenium of the corpus callosum; and cerebellar white matter (Figures 1A,B). Lesions in the areas of abnormal T2-weighted signal showed high signal intensity on diffusion-weighted imaging (DWI) without restriction on the apparent diffusion coefficient (ADC) (Figures 1C,D). No contrast enhancement was observed on the post-gadolinium T1-weighted image sequence (Figure 1E). Magnetic resonance spectroscopy was performed, and the metabolites showed normal scores in the periventricular white matter and brain stem (Supplementary Figure S1). Ophthalmological examination showed decreased mean visual acuity in patient’s left eye, despite the absence of subjective visual impairment. In the pattern visual evoked potential (VEP) test, bilateral prolonged latency and reduced amplitude of the P100 wave were detected. No chorioretinopathy or macular atrophy was observed on optical coherence tomography (OCT) examination.

Hematological testing for metabolic, infectious, and autoimmune causes was normal. Cerebrospinal fluid (CSF) analysis revealed normal results, except for slightly increased levels of protein and glucose. No oligoclonal bands (OCBs) or autoantibody markers of CNS demyelinating diseases were observed in either the serum or CSF. Electroencephalography, electromyography, and nerve conduction velocity were unremarkable.

### Identification of pathogenic variants in *CLCN2*

**FIGURE 2:**
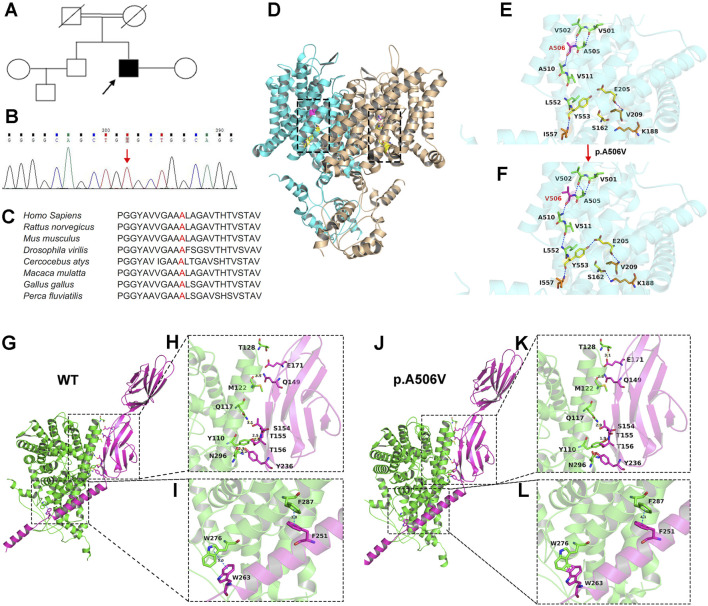
Identification of the *CLCN2* A506V variant and genetic analysis of mutant ClC-2. **(A)** Pedigree of the affected individual’s family. The patient is indicated by an arrow. **(B)** Sequencing result of the *CLCN2* variant (indicated by an arrow) in the patient. **(C)** Multiple species’ sequence alignment of the ClC-2 protein showing the high evolutionary conservation of the Ala506 amino acids. **(D)** Crystal structure of the hClC-2 homodimer (PDB code: 7XF5). Two subunits are colored cyan and wheat, respectively. Ala506 (magenta) and chloride-binding sites (yellow) are shown in the black dotted box. Only one black dotted box was shown for magnification. **(E,F)** Close-up views of wild-type hClC-2 **(E)** and A506V mutant **(F)** in cartoon representation. The positions of the substitutive amino acid residues (A506 and V506) and the surrounding amino acids (V501, V502, A505, A510, V511, and L552) are shown in sticks and are colored magenta and green, respectively. Chloride-binding sites (S162, E205, and Y553) and the surrounding amino acids (I557, K188, and V209) are shown in sticks and are colored yellow and orange, respectively. Hydrogen bonds are shown with a blue dotted line. Crystal structure of the wild-type hClC-2/GlialCAM complex **(G)** and the p.A506V mutant ClC-2/GlialCAM complex **(J)**. ClC-2 and GlialCAM are colored green and magenta, respectively. Intermolecular interactions are shown in a black dotted box. (**H,I**, **K,L**) Close-up views of the binding sites in cartoon representation. Residues involved in the intermolecular interactions are shown in sticks and are colored green (ClC-2) and magenta (GlialCAM). Yellow dotted lines represent hydrogen bonds. Blue dotted lines represent π-interactions. The lengths of non-covalent bonds are labeled on the dotted lines. All the three-dimensional models were generated using PyMOL (http://www.pymol.org). hClC-2, human ClC-2.

Given the individual’s atypical clinical presentation and imaging features on brain MRI, we performed an NGS panel of 320 candidate genes associated with leukodystrophies and genetic leukoencephalopathies. Only one missense variant, c.1517C > T (p.Ala506Val), in *CLCN2* (NM_004366.6) was identified with MAFs less than 0.05 in the patient (Figures 2A,B). The p.A506V variant in *CLCN2* was not observed in the current 1000 Genomes database and had MAFs of 0.000009 and 0.000004 in the ExAC and gnomAD databases, respectively. This variant was predicted to be pathogenic by bioinformatics analyses, including SIFT (affects protein function), PolyPhen2 (probably damaging), BayesDel_noAF (prediction score: 0.559231), REVEL (prediction score: 0.965), and VEST4 (prediction score: 0.961).

In addition, considering the clinical and radiological overlap between LKPAT and other disorders, including fragile X-associated tremor/ataxia syndrome (FXTAS) and neuronal intra-nuclear inclusion disease (NIID), which are caused by expanded trinucleotide (CGG) repeats in the 5′ untranslated region (UTR) of the *FMR1* and *NOTCH2NLC* genes, respectively, we performed gene analysis of repeat expansions in *FMR1* and *NOTCH2NLC* in our patient. The results showed that the number of GGCs in *FMR1* was 31 repeat expansions, and the *NOTCH2NLC* repeat number was 16 (Supplementary Figure S2).

### Genetic analysis

Human ClC-2 encoded by *CLCN2* is present as a homodimer (Jentsch and Pusch, 2018). Each subunit is composed of an N-terminal domain, a transmembrane helix domain (TMD), and two intracellular C-terminal cystathionine-β-synthase (CBS) domains. Each subunit also contains one independent ion pore with three chloride binding sites (S162, E205, and Y553 residues), which play a critical role in chloride ion conduction (Park et al., 2017; Ma et al., 2023). To assess the effect of the A506V variant on the ClC-2 protein structure, the predicted protein model of the A506V mutant ClC-2 was built using the SWISS-MODEL server and displayed as a crystal structural model using PyMOL. As shown in Figures 2C–F, the Ala506 amino acid is highly conserved and located in the transmembrane helix O domain near the ion channel pathway of ClC-2. A506 formed hydrogen bonds with the side chains of V502 and A510, respectively (Figure 2E). The substitution of A506 with V506 was predicted to impact the neighboring amino acid A505 and surrounding V511 and resulted in the formation of additional hydrogen bonds between A505 and V502 and between V511 and L552 (Figure 2F), which might destabilize the protein structure. Furthermore, after alteration of A506V, new interactions occurred in chloride-binding sites between E205 and Y553 and between S162 and K188 (Figure 2F). These results indicate that protein spatial conformation in the ion permeation pathway may change, and, in turn, the gating of ClC-2 is likely disrupted.

Several research studies have shown that the cell adhesion molecule GlialCAM, a ClC-2 auxiliary subunit involved in megalencephalic leukoencephalopathy with subcortical cysts (MLC), can physically bind ClC-2 and regulate its biophysical properties and subcellular localization in glial cells (Hoegg-Beiler et al., 2014; Jeworutzki et al., 2012; Gaitan-Penas et al., 2017; Passchier et al., 2024). To assess whether the A506V variant affects the interaction between ClC-2 and GlialCAM, prediction of the protein docking model was performed using the ZDOCK online server (Pierce et al., 2014). As shown in Figures 2G,J, the pose with the highest ZDOCK score was visualized and analyzed using PyMOL. Wild-type ClC-2 was bound with the extracellular immunoglobulin domain (IgC2) of GlialCAM through hydrogen bonds. Residues M122, Q117, Y110, and N296 of ClC-2 were observed to be involved in the intermolecular interactions with residues Q149, S154, T155, T156, and Y236 of GlialCAM (Figures 2G,H). In addition, wild-type ClC-2 residues F287 and W276 formed π-interactions with GlialCAM residues F251 and W263, respectively (Figures 2G,I). However, the A506V variant contributed to changes in intermolecular interactions (Figures 2J–L). A new hydrogen bond was formed between T128 and E171, and other hydrogen bond lengths decreased. Moreover, covalent interaction was observed at Y110–S154, Y110–T156, N296–Y236, and W276–W263 instead of hydrogen bonds.

### Literature review

**FIGURE 3:**
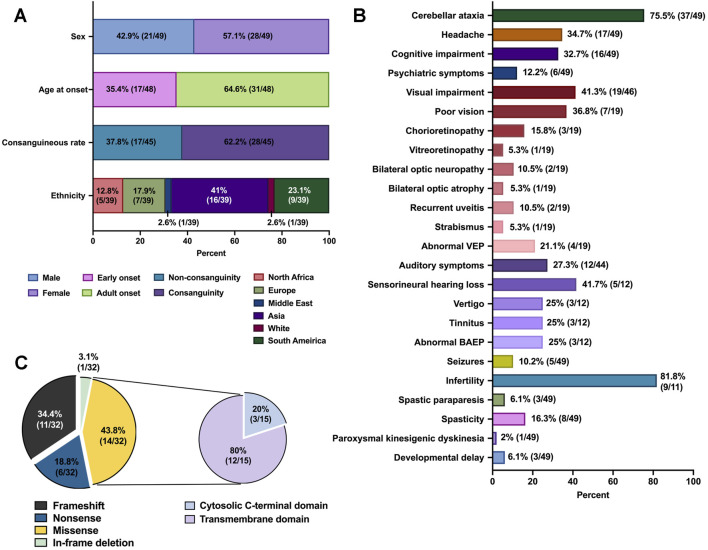
Clinical and genetic characteristics of the reported patients with LKPAT. **(A)** Percentage distribution of demographic information in LKPAT patients. **(B)** Percentage distribution of clinical features in LKPAT patients. **(C)** Percentage distribution of different types of *CLCN2* pathogenic variants identified in LKPAT patients. The subgraph shows the distribution of positions among the total missense variants and in-frame deletion.

Through a literature review, we identified 49 LKPAT patients previously described, including 21 male individuals (42.9%) and 28 female individuals (57.1%) (Figure 3A; Supplementary Table S1) (Depienne et al., 2013; Di Bella et al., 2014; Hanagasi et al., 2015; Giorgio et al., 2017; Zeydan et al., 2017; Hoshi et al., 2019; Guo et al., 2019; Ozaki et al., 2020; Parayil Sankaran et al., 2020; Ngo et al., 2020; Shi et al., 2021; Ben Mohamed et al., 2022; Abreu et al., 2023; Almasoudi et al., 2023; Holla et al., 2023; Wu et al., 2023; Cheng et al., 2023; Nóbrega et al., 2024; Garg et al., 2024; Zhou et al., 2025; Ohira et al., 2024; Orimo et al., 2025; Bavdhankar et al., 2025). The average age of onset was 27 ± 20 (mean ± SD) years (between 3 months and 69 years). The proportion of adult-onset LKPAT was 64.6% (31/48). The consanguineous rate was 62.2% (28/45). Prevalence estimates vary across geographical regions. The frequency of LKPAT was estimated to be the highest in Asia (16/39, 41%, particularly in Japan and China). LKPAT patients exhibited wide phenotypic variability (Figure 3B). The most frequent phenotype was cerebellar ataxia (37/49, 75.5%). Visual impairment, the second most common manifestation, was present in 19/46 cases (41.3%), followed by poor vision (7/19, 36.8%), chorioretinopathy (3/19, 15.8%), vitreoretinopathy (1/19, 5.3%), bilateral optic neuropathy (2/19, 10.5%), bilateral optic atrophy (1/19, 5.3%), recurrent uveitis (2/19, 10.5%), strabismus (1/19, 5.3%), angle closure glaucoma (1/19, 5.3%), and abnormal VEP (4/19, 21.1%). Other variable neurological features included headache (17/49, 34.7%), cognitive impairment (16/49, 32.7%), psychiatric symptoms (6/49, 12.2%), spasticity (8/49, 16.3%), and spastic paraparesis (3/40, 7.5%). Auditory symptoms were reported in 27.3% of cases (12/44) involving sensorineural hearing loss (5/12, 41.7%), vertigo (3/12, 25%), tinnitus (3/12, 25%), and abnormal BAEP (3/12, 25%). Seizures were present in 5/49 cases (10.2%), and two of them were tonic–clonic seizures. Rare symptoms included paroxysmal kinesigenic dyskinesia, paraparesis, myoclonus, pyramidal signs, numbness, back pain, developmental delay, borderline macrocephaly, ptosis, and hyperthyroidism. Notably, among the adult males, 4/11 cases (36.4%) included infertility due to azoospermia, and three other male patients (5/11, 45.5%) involuntarily had no offspring, suggesting possible infertility.

Furthermore, we reviewed that 32 pathogenic variants of *CLCN2* including the one we reported have been identified in LKPAT patients (Figure 3C; Supplementary Table S1). A total of 11 pathogenic variants (34.4%) were frameshift variants, and 6 variants (18.8%) were nonsense variants. In addition, there were 14/32 (43.8%) missense variants and 1/32 in-frame deletion (3.1%) in LKPAT patients.

## Discussion

LKPAT was first reported in six unrelated patients who presented with variable clinical features and harbored biallelic *CLCN2* variants (Depienne et al., 2013). So far, only 49 individuals with LKPAT, including our patient, have been described in detail (Depienne et al., 2013; Di Bella et al., 2014; Hanagasi et al., 2015; Giorgio et al., 2017; Zeydan et al., 2017; Hoshi et al., 2019; Guo et al., 2019; Ozaki et al., 2020; Parayil Sankaran et al., 2020; Ngo et al., 2020; Shi et al., 2021; Ben Mohamed et al., 2022; Abreu et al., 2023; Almasoudi et al., 2023; Holla et al., 2023; Wu et al., 2023; Cheng et al., 2023; Nóbrega et al., 2024; Garg et al., 2024; Zhou et al., 2025; Ohira et al., 2024; Orimo et al., 2025; Bavdhankar et al., 2025). The small number of known affected individuals may be attributed to high phenotypic variability and a mild clinical course, which may result in delayed diagnosis. Understanding the clinical characteristics and genotype–phenotype correlation is helpful for the diagnosis, genetic counseling, and development of therapeutics for LKPAT.

In this study, we reviewed the clinical and genetic features of all affected individuals reported from 2013 to date. We observed that LKPAT patients presented with a broad range of ages of onset, and adult onset was more common. Although LKPAT patients exhibited heterogeneous clinical manifestations, our literature review indicates that the majority of patients presented with ataxia. Other frequent features, including visual impairment, auditory deficits, headache, and cognitive dysfunction, were observed. Notably, most male individuals were confirmed to be infertile or failed to reproduce. Therefore, visual and auditory impairments, along with male infertility, may serve as distinctive clinical indicators of LKPAT. In this study, our patient showed atypical neurological features, including dizziness, monoparesis, and mild cerebellar ataxia. He denied any visual complaints, while the VEP test results were abnormal. In particular, he had a history of azoospermia. These characteristics were suggestive findings of LKPAT and prompted evaluation by brain MRI. In previous research studies, all cases with LKPAT showed a typical imaging appearance on brain MRI that included abnormally symmetrical and confluent hypointense signals on T1-weighted images and hyperintense signals on T2-weighted images, particularly in the posterior limbs of the internal capsules, midbrain cerebral peduncles, and middle cerebellar peduncles (Depienne et al., 2013; Guo et al., 2019). Lesions can also be observed in the central tegmental tracts, pyramidal tracts in the pons, cerebral white matter, corpus callosum, and cerebellar white matter (Nóbrega et al., 2024). LKPAT patients showed DWI hyperintensities, with or without diffusion restriction, in the affected white matter. The ADC values are associated with the size of myelin vacuoles and extracellular spaces (Depienne et al., 2013; van der Voorn et al., 2006). In our patient, the increased ADC values may be due to large vacuoles and expanded intra-myelinic water spaces in the lesions. Notably, although symmetric lesions were detected through brain MRI, some LKPAT cases, including our patient, presented with unilateral symptoms, manifesting as left-sided limb weakness, right-sided numbness, spastic paralysis of the right arm, or mild ataxia in the left upper extremity (Zeydan et al., 2017; Almasoudi et al., 2023). Accumulating pieces of evidence have indicated that the MRI changes precede the onset of neurological symptoms in LKPAT, accompanied by an extremely slow disease progression (Di Bella et al., 2014; Giorgio et al., 2017; Guo et al., 2019). As the disease progresses, patients may subsequently develop corresponding symptoms in the contralateral limbs (Almasoudi et al., 2023). In our study, the patient’s clinical manifestations may partially be attributed to the early disease stage, and bilateral weakness is likely to manifest with disease progression, for which long-term follow-up is required.

The specific phenotype (for example, ataxia, visual impairment, and male infertility) combined with highly typical imaging abnormalities may be a strong diagnostic clue of LKPAT. Notably, it is important to consider FXTAS and NIID as differential diagnoses due to the overlapping phenotypes with LKPAT (Jacquemont et al., 2003; Sone et al., 2019). FXTAS is an X-linked neurodegenerative disorder caused by short CGG expansion (55–200 repeats) in the UTR of the *FMR1* gene (Jacquemont et al., 2003; Verkerk et al., 1991). Similar to LKPAT, the core clinical manifestations of FXTAS are intention tremor and cerebellar ataxia (Jacquemont et al., 2003). However, FXTAS is a late-onset disorder usually presenting at the mean age of 62 and commonly manifests with executive dysfunction, Parkinsonism, and neuropathy (Cabal-Herrera et al., 2020). Although brain MRI of FXTAS typically shows symmetrical white matter abnormalities with hyperintense signals on T2-weighted images in the middle cerebellar peduncles, no signal abnormalities have been reported in the posterior limbs of the internal capsules and midbrain cerebral peduncles (Cohen et al., 2006; Filley et al., 2015). Moreover, generalized brain atrophy, corpus callosum thinning, and ventricular enlargement are also present in patients with FXTAS, which are not observed in LKPAT cases (Cohen et al., 2006; Filley et al., 2015; Hashimoto et al., 2011). Thus, FXTAS can be differentiated from LKPAT by these features. NIID is a rare neurodegenerative disease caused by expanded GGC repeats in the *NOTCH2NLC* gene (Sone et al., 2019). NIID and LKPAT share overlapping clinical features, including ataxia, cognitive impairment, muscle weakness, and seizures, which cause diagnostic confusion in the early stages (Sone et al., 2016). However, NIID typically shows bilateral hyperintensity at the corticomedullary junction on DWI images, a feature that is absent in LKPAT (Nóbrega et al., 2024; Garg et al., 2024; Sone et al., 2016; Yu et al., 2019). In this study, the results of *FMR1* and *NOTCH2NLC* gene analysis in our patient showed normal repeat expansions. Based on the patient’s clinical features and MRI findings, the diagnoses of FXTAS and NIID can both be excluded.

**FIGURE 4:**
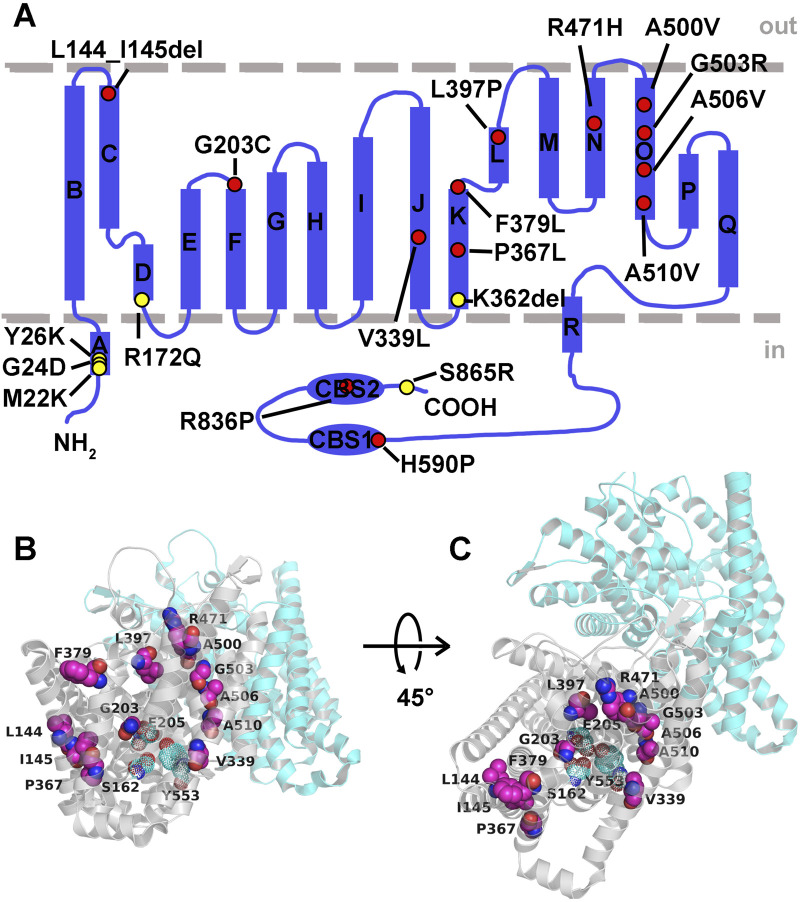
Structure of hClC-2 and residues affected by pathogenic variants in *CLCN2*-related diseases. **(A)** Overall topology of hClC-2 with 18 α-helices (labeled as A-R) and two CBS domains. The positions of the *CLCN2* pathogenic variants identified in LKPAT and FH-II are labeled as red and yellow dots, respectively. **(B,C)** Two views of three-dimensional structure of the transmembrane domains of hClC-2 homodimer (PDB code: 7XF5). Two subunits are colored cyan and gray, respectively. Amino acid residues of the pathogenic variants and chloride-binding sites are shown in magenta spheres and cyan dots, respectively. CBS, cytosolic cystathionine beta synthase; hClC-2, human ClC-2.

The definitive diagnosis of LKPAT is confirmed by the identification of biallelic pathogenic variants in *CLCN2* through molecular genetic testing. The ClC-2 protein is expressed in the plasma membrane, where it transports chloride ions and performs critical functions in cellular homeostasis (Thiemann et al., 1992; Wang et al., 2017). Each subunit of the ClC-2 homodimer is composed of 18 α-helices (named helix A-R) and 2 CBS domains (Figure 4A). Helix B–R within the transmembrane domain containing an ion transport pathway is involved in chloride conduction, and the CBS domains play a modulating role associated with voltage-dependent channel activation (Wang et al., 2017; Yusef et al., 2006). At present, a total of 32 pathogenic missense variants of *CLCN2* in LKPAT patients have been described in the literature. More than half of the variants were truncation variants, which can cause premature stop codons and consequently lead to nonsense-mediated mRNA decay or protein truncation (Jentsch and Pusch, 2018). These pathogenic variants may directly decrease ClC-2 expression or affect the formation of a functional channel, thereby leading to loss of ClC-2 function, which is closely associated with LKPAT (Jentsch and Pusch, 2018; Gaitan-Penas et al., 2017). In addition, nearly half were missense variants, most of which were located in the transmembrane domain around the ion permeation pathway of ClC-2 (Figures 4A–C). Recently, researchers have identified three chloride-binding sites along the channel in human ClC-2, including pore filter residues S162 and Y553 and gating glutamate E205 (Ma et al., 2023; McKiernan et al., 2020). The opening and closing of the ClC-2 transporter are controlled by the gating glutamate through swinging the side chain of E205, which regulates chloride ion selectivity and permeability (Park et al., 2017; Dutzler et al., 2003). In the present study, we reported a novel homozygous *CLCN2* A506V variant located near the ion permeation pathway and analyzed its potential pathogenic mechanism through *in silico* analysis. We observed that substitution of A506 with V506 altered hydrogen bond formation in nearby amino acids, particularly in chloride-binding sites in the crystal structural model of human ClC-2. As hydrogen bonds are important for protein spatial conformation and stability (Cao et al., 2024; Dong et al., 2022), the subtle changes between E205 and Y553 and between S162 and K188 may interfere with chloride conduction and impair the gating properties of ClC-2. Furthermore, GlialCAM, as a ClC-2-binding partner, has been reported to target ClC-2 to astrocyte–astrocyte junctions and glia limitans in white matter, stabilize ClC-2 expression at the plasma membrane, and increase its channel activity (Jeworutzki et al., 2012; Gaitan-Penas et al., 2017). Reduced ClC-2 expression in the plasma membrane and chloride currents caused by the *CLCN2* A500V variant located around the ion permeation pathway can be partially rescued by co-expression with GlialCAM in glial cells (Gaitan-Penas et al., 2017). In this study, our results showed that hydrogen bonds and π-interactions between the interface residues of ClC-2 and GlialCAM were changed in the predicted protein interaction model by the ZDOCK program. This altered interaction of ClC-2 and GlialCAM may contribute to the abnormal formation of a complex and disrupt the stability and biophysical properties of ClC-2. Therefore, we speculate that the *CLCN2* A506V variant located around the ion permeation pathway may have a negative effect on chloride conduction and GlialCAM–ClC-2 interaction in glial cells, which fail to compensate for an action-potential-induced excess of potassium in myelinated axons and, consequently, lead to osmotic intra-myelinic edema (Goppner et al., 2021; Blan et al., 2007; Rash, 2010). Moreover, impaired transportation of chloride ions may change the ionic environment of germ cells, thus causing testicular degeneration and infertility (Blan et al., 2007; Bosl et al., 2001).

Notably, some previously reported missense variants have been shown to cause gain-of-function of ClC-2 channels, which is associated with familial hyperaldosteronism type II (FH-II) (Scholl, 2022). In contrast to the loss-of-function *CLCN2* variants, all pathogenic variants identified in patients with FH-II so far are located in the cytosolic domains (predominantly in the N-terminal inactivation domain) or near the cytosolic ends of transmembrane helices that are hypothesized to interact with the N-terminal inactivation domain of ClC-2 (Jordt and Jentsch, 1997) (Figure 4A). These missense variants could open the ClC-2 channel, thus resulting in increased chloride efflux and continuous depolarization in zona glomerulosa cells (Jordt and Jentsch, 1997; Scholl et al., 2018; Stowasser et al., 2019; Grunder et al., 1992). Cellular depolarization activates voltage-dependent calcium channels, enhances calcium influx, and ultimately increases aldosterone production by inducing the expression of aldosterone synthase, a rate-limiting enzyme for aldosterone biosynthesis (Scholl et al., 2018; Stowasser et al., 2019). Therefore, we provide the potential genotype–phenotype features of *CLCN2*-related diseases as follows: *CLCN2* pathogenic variants identified in human beings, in the form of loss-of-function and gain-of-function variants, are associated with LKPAT and FH-II, respectively. LKPAT-related pathogenic variants are mostly located in transmembrane domains near the chloride ion transport pathway or cause truncated proteins, whereas variants of FH-II mainly affect the N-terminal inactivation domain. However, a broad range of ages of onset and phenotypic variability were observed among individuals with LKPAT carrying the same *CLCN2* variants. Further studies are needed to analyze the genotype–phenotype correlation of LKPAT. A limitation of this study is the lack of family validation. We conducted a combination of multiple bioinformatics and functional analyses to provide reliable evidence supporting the pathogenicity of the identified variant. In-depth mechanistic studies are required to analyze the effect of the A506V variant in *CLCN2*-related leukoencephalopathy.

## Conclusion

We identified a novel *CLCN2* homozygous pathogenic variant in a patient presenting with LKPAT. Slow progression and non-specific and highly variable clinical symptoms make the diagnosis of *CLCN2*-related leukoencephalopathy challenging. Individuals with ataxia, auditory or visual impairment, and male individuals with infertility, coupled with the characteristic white matter changes in MRI, should be tested for *CLCN2*-related leukoencephalopathy. Currently, there are no specific treatments for this disease. Prenatal genetic testing is essential to prevent the birth of affected children. Our study expands the genotypic spectrum of LKPAT, indicates the potential pathogenesis of the *CLCN2* A506V variant, and provides valuable insights for further studies toward the therapeutics of *CLCN2*-related leukoencephalopathy.

## Data Availability

The original contributions presented in the study are included in the article/Supplementary Material, further inquiries can be directed to the corresponding author.
