# Supplementary material for: Identification of a novel CLCN2 homozygous variant in a man with leukoencephalopathy and infertility: a case report and literature review
Source: Front Genet. 2026 Feb 25;17:1761205. doi: 10.3389/fgene.2026.1761205 (PMC12975440; doi:10.3389/fgene.2026.1761205)
Supplement: Supplementary file 1 [file DataSheet1.doc]

Table S1. Clinical and genetic features of the reported patients with *CLCN2*-related leukoencephalopathy

| **Patients** | **Ref** | ***CLCN2* mutations** | **Genotype** | **Location** | **Ethnicity** | **Consanguinity** | **Sex** | **Age of onset** | **Neurological features** | **Auditory symptoms** | **Visual impairment** | **Male** **infertility** | **Other features** |
| --- | --- | --- | --- | --- | --- | --- | --- | --- | --- | --- | --- | --- | --- |
| 1 | [2] | c.1709G>A (p.Trp570*) | Hom | CTD (loop between R-CBS1~) | North Africa | - | F | 44y | Mild ataxia | - | - | NA | - |
| 2 | [2] | c.1709G>A (p.Trp570*) | Hom | CTD (loop between R-CBS1~) | North Africa | - | F | 57y | Mild ataxia | Tinnitus, vertigo, deafness | Retinoschisis, bilateral optic neuropathy | NA | - |
| 3 | [2] | c.430_435del (p.Leu144_Ile145del) | Hom | TMD (helix C) | North Africa | + | F | 30y | Mild ataxia, psychosis, severe cognitive impairment, headache | - | Chorioretinopathy | NA | - |
| 4 | [2] | c.1143delT (p.Gly382Alafs*34);  c.64–1107_639del (p.Met22Leufs*5) | Het  Het | TMD (loop between K-L~)  NTD | Europe | ND | F | 12y | Mild ataxia, mild cognitive impairment, severe headache | - | - | NA | Mild spasticity |
| 5 | [2] | c.1499C>T (p.Ala500Val) | Hom | TMD (helix O) | Europe | - | M | 6y | Mild ataxia, severe headache | - | - | ND | - |
| 6 | [2] | c.828dupG (p.Arg277Alafs*23) | Hom | TMD (helix I~) | Europe | + | F | 3y | Mild ataxia, mild cognitive impairment | - | - | NA | Mild spasticity |
| 7 | [3] | c.1507G>A (p.Gly503Arg) | Hom | TMD (helix O) | Europe | + | M | 42y | Mild ataxia | Abnormal BAEP | Abnormal ERG and VEP | + | Borderline macrocephaly |
| 8 | [4] | c.1113delinsACTGCTCAT (p.Ser375Cysfs*6) | Hom | TMD (helix K~) | Middle East | + | F | 7y | Mild ataxia, mild cognitive impairment | - | - | NA | PKD |
| 9 | [5] | c.1769A > C (p.His590Pro) | Hom | CTD (CBS1) | North Africa | - | F | 52y | Mild ataxia, moderate headache | Abnormal BAEP | Mild and asymptomatic bilateral optic atrophy | NA | - |
| 10 | [6] | c.1412G>A (p.Arg471His) | Hom | TMD (helix N) | ND | + | F | 28y | Moderate ataxia | Unilateral mild conductive type hearing loss | - | NA | - |
| 11 | [6] | p.Glu475Lysfs*79 | Hom | TMD (helix N~) | ND | + | F | 27y | Mild ataxia, headache | - | Blurry vision | NA | Paraparesis |
| 12 | [6] | p.Leu435Argfs*7 | Com.Het | TMD (helix M~) | ND | - | F | 46y | Mild ataxia | - | - | NA | Numbness |
| 13 | [7] | c.61dupC  (p.Leu21Profs*27) | Hom | NTD | Asia (Japan) | - | F | 3m | - | - | - | NA | Tonic-clonic seizure |
| 14 | [8] | c.2257C > T (p.Arg753*) | Hom | CTD (loop between CBS1-CBS2~) | ND | + | F | 22y | Mild ataxia, mild memory decline | Mild tinnitus, dizziness | Poor vision | NA | Hyperthyroidism |
| 15 | [9] | c.61dupC (p.Leu21Profs*27) | Hom | NTD | ND | - | M | 6y | - | - | - | ND | Aseptic meningitis |
| 16 | [10] | c.1100C>T (p.Pro367Leu) | Hom | TMD (helix K) | ND | ND | M | 11y | Episodic headache | Sensorineural hearing loss, vertigo | - | ND | - |
| 17 | [11] | c.1153C>T (p.Gln385*) | Hom | TMD (loop between K-L~) | White (US) | ND | F | Adult-onset | Ataxia | ND | ND | NA | - |
| 18 | [12] | c.211C>T  (P.Arg71*) | Hom | NTD | Asia (China) | - | F | 48y | Ataxia, cognitive impairment, headache | ND | ND | NA | Pyramidal sign |
| 19 | [13] | c.1709G>A (p.Trp570*) | Hom | CTD (loop between R-CBS1~) | North Africa | + | F | 45y | Mild ataxia, psycho-cognitive disturbances, tension-type headache | Tinnitus | - | NA | Mild spasticity |
| 20 | [14] | c.1709G>A (p.Trp570*) | Hom | CTD (loop between R-CBS1~) | ND | - | M | 2y | Mild ataxia | - | Potential involvement of the optic pathway | ND | - |
| 21 | [15] | c.607G > T (p.Gly203Cys) | Hom | TMD (loop between E-F) | Asia (Iraqi) | + | F | 6m | Mild ataxia, cognitive impairment | ND | Visual dysfunction | NA | Spastic paraplegia |
| 22 | [15] | c.607G > T (p.Gly203Cys) | Hom | TMD (loop between E-F) | Asia (Iraqi) | + | M | 6y | Mild ataxia, cognitive impairment | ND | Chorioretinopathy | + | Spastic paraplegia |
| 23 | [15] | c.607G > T (p.Gly203Cys) | Hom | TMD (loop between E-F) | Asia (Iraqi) | + | M | ND | Mild forgetfulness | ND | ND | + | - |
| 24 | [16] | 1542_1543delAG (p.Ser516Hisfs*38) | Hom | TMD (helix P~) | ND | + | F | 18y | Mild ataxia, mild-moderate headache | Dizziness, abnormal BAEP | Abnormal VEP | NA | Mild spasticity |
| 25 | [16] | c.1137C>A (p.Phe379Leu) uncertain | Hom | TMD (loop between K-L) | ND | + | M | 29y | Mild ataxia, mild-moderate headache | - | - | - | Mild spasticity |
| 26 | [17] | c.2507G>C (p.Arg836Pro) | Hom | CTD (CBS2) | Asia (China) | ND | M | 41y | Severe headache | - | - | ND | - |
| 27 | [18] | c.1382_1386del (p.Pro461Leufs*13) | Hom | TMD (helix N~) | Asia (China) | - | F | 18y | - | - | Vitreoretinopathy | NA | - |
| 28 | [19] | c.1709G>A (p.Trp570*) | Hom | CTD (loop between R-CBS1~) | South America | + | M | 13y | - | - | - | NA | Spastic paraparesis |
| 29 | [19] | c.1709G>A (p.Trp570*)  c.1529C>T (p.Ala510Val) | Com.Het | CTD (loop between R-CBS1~)  TMD (helix O) | South America | - | F | 30y | - | - | - | NA | Ptosis |
| 30 | [19] | c.1709G>A (p.Trp570*) | Hom | CTD (loop between R-CBS1~) | South America | + | M | 8y | Ataxia | - | - | NA | Pyramidal signs |
| 31 | [19] | c.1529C>T (p.Ala510Val) | Hom | TMD (helix O) | South America | + | M | 6m | Ataxia, autism spectrum disorder | - | Poor visual contact | NA | Developmental delay, ptosis |
| 32 | [19] | c.1709G>A (p.Trp570*) | Hom | CTD (loop between R-CBS1~) | South America | + | M | 54y | Ataxia | - | Visual impairment, recurrent uveitis | Possible | - |
| 33 | [19] | c.1190T>C (p.Leu397Pro) | Hom | TMD (loop between L-M~) | Europe | + | F | 18y | - | - | - | NA | Back pain |
| 34 | [19] | c.1412G>A (p.Arg471His) | Hom | TMD (helix N) | Europe | + | F | 47y | Ataxia, subtle memory impairment, headache | - | - | NA | Tonic-clonic seizures, myoclonus |
| 35 | [19] | c.2438delT (p.Leu813Argfs*20) c.1709G>A (p.Trp570*) | Com.Het | CTD (CBS2)  CTD (loop between R-CBS1~) | South America | - | M | 62y | Ataxia, cognitive impairment, behavior changes | - | - | Possible | Seizures, hyper-reflexia |
| 36 | [19] | c.1709G>A (p.Trp570*) | Hom | CTD (loop between R-CBS1~) | South America | - | F | 14y | Ataxia, cognitive decline, headache | - | - | NA | Seizures, paroxysmal dyskinesia |
| 37 | [19] | c.1015G>C (p.Val339Leu) | Hom | TMD (helix J) | Europe | + | F | 36y | Ataxia, psychosis | - | - | NA | - |
| 38 | [19] | c.1709G>A (p.Trp570*) | Hom | CTD (loop between R-CBS1~) | South America | + | M | 3y | Ataxia, moderate intellectual disability | - | Strabismus | NA | Developmental delay |
| 39 | [19] | c.1709G>A (p.Trp570*) | Hom | CTD (loop between R-CBS1~) | South America | + | M | 26y | Ataxia | - | - | Possible | - |
| 40 | [20] | c.1651_1652dupTC  (p.Leu552fs*64) | Hom | TMD  (helix R~) | ND | + | F | 18y | Ataxia | - | - | NA | lower limb spasticity |
| 41 | [21] | c.2201dup  (p.Glu735Ter) | Hom | CTD (loop between CBS1-CBS2) | Asia (China) | + | M | 7m | Borderline development | - | - | ND | Epileptic seizures |
| 42 | [22] | c.1828 C>T  (p.Arg610*)  c.61dupC  (p.Leu21Profs*27) | Com.Het | CTD (CBS1)    NTD | Asia (Japan) | - | F | 31y | Mild headache | vertigo | Angle closure glaucoma | NA | Hyper-reflexia |
| 43 | [23] | c.61dupC  (p.Leu21Profs*27)  c.983 +2 T>A | Com.Het | NTD  TMD (helix J~) | Asia (Japan) | - | M | 57y | Ataxia | Deafness | VEP P100 prolongation | - | Hyper-reflexia |
| 44 | [23] | c.61dupC  (p.Leu21Profs*27) | Hom | NTD | Asia (Japan) | - | F | 69y | Ataxia, headache | - | Visual impairment | NA |  |
| 45 | [23] | c.61dupC  (p.Leu21Profs*27)  c.1828 C>T  (p.Arg610*) | Com.Het | NTD  CTD (CBS1) | Asia (Japan) | - | M | 32y | Headache | Deafness | Visual impairment, uveitis | Possible | Spasticity, hyper-reflexia |
| 46 | [23] | c.61dupC  p.(Leu21Profs*27) | Hom | NTD | Asia (Japan) | + | M | 62y | Ataxia, cognitive dysfunction | - | - | Possible | Hyper-reflexia |
| 47 | [24] | C1672C > T  (p.Arg558Ter) | Hom | TMD (helix R) | Asia (India) | + | F | 8y | Mild ataxia | - | - | NA | Dystonia, dystonic tremor, myoclonic jerks |
| 48 | [24] | c.1412G>A (p.Arg471His) | Hom | TMD (helix N) | Asia (India) | + | F | 32y | Ataxia, occipital headaches, cognitive dysfunction, severe depression | - | - | NA | Spasticity |
| 49 |  | c.1517C>T (p.Ala506Val) | Hom | TMD  (helix O) | Asia (China) | + | M | 45y | Mild ataxia | Dizziness | Abnormal VEP | + | - |

TMD, transmembrane domain, CTD, cytosolic C-terminal domain; VEP, visual evoked potentials; BAEP, brainstem auditory evoked potentials; PKD, paroxysmal kinesigenic dyskinesia; NA, information not available; ND, not disclosed; ~, truncated protein., cataract, vitreomacular traction syndrome, Macular edema, right epiretinal membrane


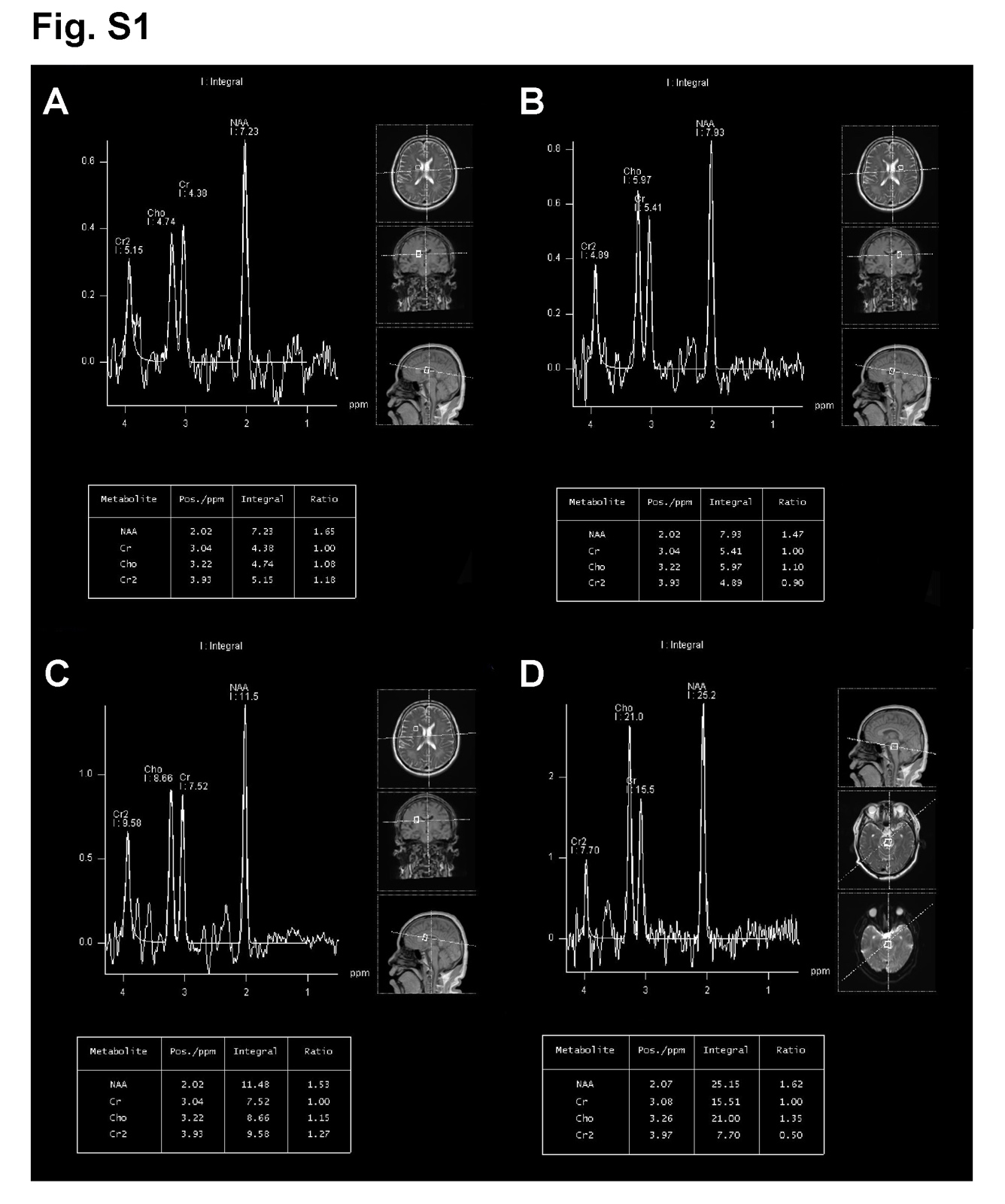


**Fig S1.** Magnetic resonance spectroscopy was performed in the periventricular white matter and brain stem of our patient.


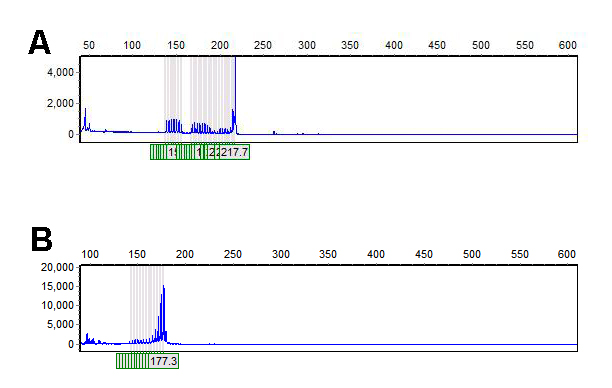


**Fig S2.** Gene analysis of repeat expansions in *FMR1* (A) and *NOTCH2NLC* (B) in our patient.
